# Working with Complex Needs in Rehabilitation Services and Beyond – Evaluating a Bespoke Training Programme to Support Workforce Development

**DOI:** 10.1192/bjo.2026.11335

**Published:** 2026-06-30

**Authors:** Jordan Burrell, Amrith Shetty, Rajan Nathan

**Affiliations:** 1 Cheshire & Wirral Partnership NHS Foundation Trust, Chester, United Kingdom; 2 University of Chester, Chester, United Kingdom; 3 Liverpool John Moores University, Liverpool, United Kingdom

## Abstract

**Aims:**

Investing in workforce development is a core tenet of delivering high quality, safe and effective mental health care, particularly when supporting individuals with long-term and complex needs. Further to preliminary piloting and evaluating of a novel training package to support clinical staff to deliver values-led, recovery orientated rehabilitation, we have revised the package and commenced a wider evaluation. The aim of this study was to deliver a revised module to a wider audience and analyse its impact on participants using quantitative and qualitative methods.

**Methods:**

The “Collaborating Towards Recovery” module was offered to staff working in mental health services across the North West of England. This module was selected as the content is focused more on general principles of recovery orientated mental health care,rather than being solely focused on rehabilitation. Participants were recruited via expressions of interest through established practice networks.

Data was collected through pre-and post-training questionnaires, alongside a focus group facilitated 6 weeks post-training. All questionnaires were anonymous and completion of both questionnaires and attendance at the focus group was optional.

Qualitative data was collated, and a reflexive thematic analysis was undertaken to identify key themes.

**Results:**

Of the n=12 participants who completed the post-training questionnaire, the majority identified their role as clinical, with a small number of participants stating their role was operational (n=1) or lived experience (n=1). Self-rated knowledge and understanding of recovery approaches increased from an average of 6.4/10 (median=7) to 8.7/10 (median=9) on completion of the training module. Overall, 100% of post-training respondents felt the training was relevant to them, alongside 100% of respondents reporting feeling they could put the themes discussed into practice.

Thematic analysis of the qualitative data identified six key themes: (1) shaping new perspectives, (2) learning through connection, (3) flexible facilitation, (4) balance of philosophy and practice, (5) reframing needs and holding hope and (6) hearing lived experience voices.

**Conclusion:**

The data reflects that participants, overall, had a positive experience of participating in the training. Not only did the training enhance self-rated knowledge and understanding of recovery, but feedback reflecting the value of connection, inclusion of lived experience voices and fostering and holding hope gives evidence that recognises the overallvalue of the training module. Feedback also reflects areas for improvement or further evaluation, including the amount of content delivered, alongside further considerations for the practical application of the principles in different areas of practice.

